# Preface to “Aziridine Chemistry”

**DOI:** 10.3390/molecules26061525

**Published:** 2021-03-11

**Authors:** Hyun-Joon Ha

**Affiliations:** Department of Chemistry, Hankuk University of Foreign Studies, Yongin, Kyunggi-Do 17035, Korea; hjha@hufs.ac.kr

Aziridine is a nitrogen-containing three-membered ring with similar ring strain energy as other three-membered ring compounds, including cyclopropane and oxirane [1,2]. Its high ring strain energy renders various nitrogen-containing cyclic and acyclic compounds as oxirane (epoxide) does to realize oxygen-containing compounds as shown in Figure 1 [3]. 

The central biomolecular structure of genetic material is composed of nitrogen-based molecules with a close chemical relationship between elementary carbon and nitrogen [4]. Unless we are not able to change the atom in one specific molecule, nitrogen is one of the essential and most abundant atoms in valuable molecules, including natural products and drugs. Many drugs and their candidates contain amines in medicinal chemistry. Building these amine-containing molecules sometimes requires multiple steps. Some of these nitrogen-containing molecules may be realized by a shortcut with the formation of aziridine as a starting material or an advanced synthetic intermediate [5]. Many synthetic organic chemists have explored “aziridine chemistry” including efficient synthesis of valuable aziridines and their utilization as key synthetic intermediates in cooperation with nitrogen. However, aziridine has been studied relatively less extensively. It has been used in limited cases compared to oxirane(epoxide) because its preparation and utilization are relatively arduous. 

According to Scopus^®^, the number of publications with the search term “aziridine” is 6925 from 1948 to 1 October 2020, which is less than 13% compared to the numbers with the search term “oxirane” (or “epoxide”). Aziridine was called the “Ugly-Cousin of Epoxide” by Sweeny in his review article [6] compared to three-member heterocycle epoxide with similar ring strain. However, the chemistry of aziridine is sometimes laborious with some difficulties to carry on. Its handling is also quite messy to deal with. Amine is trivalent with the formation of three different bonds, at which points aziridine is more complicated compared to divalent epoxide. In addition, amine is more nucleophilic and more reactive than alcohols and water. Reactions of amines with acyl derivatives are faster than corresponding transformations with alcohols.

The stability and diversity of aziridines including ring-opening by nucleophiles are dependent on substituents at the ring nitrogen (whether they are electron-withdrawing or electron-donating) [7,8]. Aziridines are bifurcated into “activated” ones bearing electron-withdrawing substituents at the ring nitrogen and “non-activated” ones with electron-donating substituents. Successful tailing of aziridines with various substituents would warrant a streamlined synthesis of valuable and biologically active amines and heterocycles by regio- and stereoselective aziridine-ring openings and ring transformations [9,10].

Like the fairytale story “Beauty and the Beast”, a curse of an Enchantress made a handsome prince into a terrible beast because of his selfishness. He should find true love before the last rose petal falls. We are able to adopt this story to aziridine, with more difficulties in carrying out its chemistry and handling compared to epoxide. We have to be a good chemist to overcome the difficulties of aziridine as Belle did with the selfish beast, for the prince came back to his original handsome figure. With good tools to handle aziridine, including its efficient preparation and chemical transformation, aziridine can serve as a valuable molecule containing the essential nitrogen element in cyclic and acyclic forms. To change “the Ugly Aziridine” to “the Beauty Aziridine”, various chemical work is required. From the point of view this Special Issue, “aziridine chemistry” is needed more than ever for the development of valuable nitrogen-compounds. This Special Issue includes several communications in the form of original research and review articles covering aziridine synthesis and its utilization as starting materials and synthetic intermediates. Within the scope of “aziridine chemistry”, I would like to introduce you to this Special Issue of “the Beauty Aziridine” 133 years after Gabriel’s discovery of aziridine in 1888 [11].

## Figures and Tables

**Figure 1:**
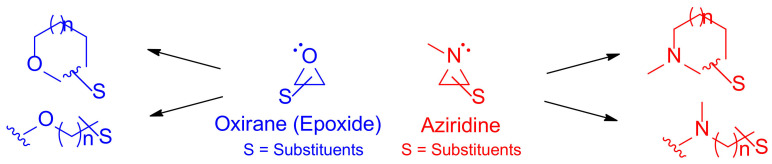
Oxirane (epoxide) yields cyclic and/or acyclic compounds with oxygen in blue, while aziridine and its transformations toward cyclic and/or acyclic nitrogen-containing molecules are shown in red.
